# Supplementary material for: Engineering the haemogenic niche mitigates endogenous inhibitory signals and controls pluripotent stem cell-derived blood emergence
Source: Nat Commun. 2017 May 25;8:15380. doi: 10.1038/ncomms15380 (PMC5477512; doi:10.1038/ncomms15380)
Supplement: Supplementary Information — Supplementary Figures and Supplementary Table [file ncomms15380-s1.pdf]

SUPPLEMENTARY INFORMATION

Supplementary Figures

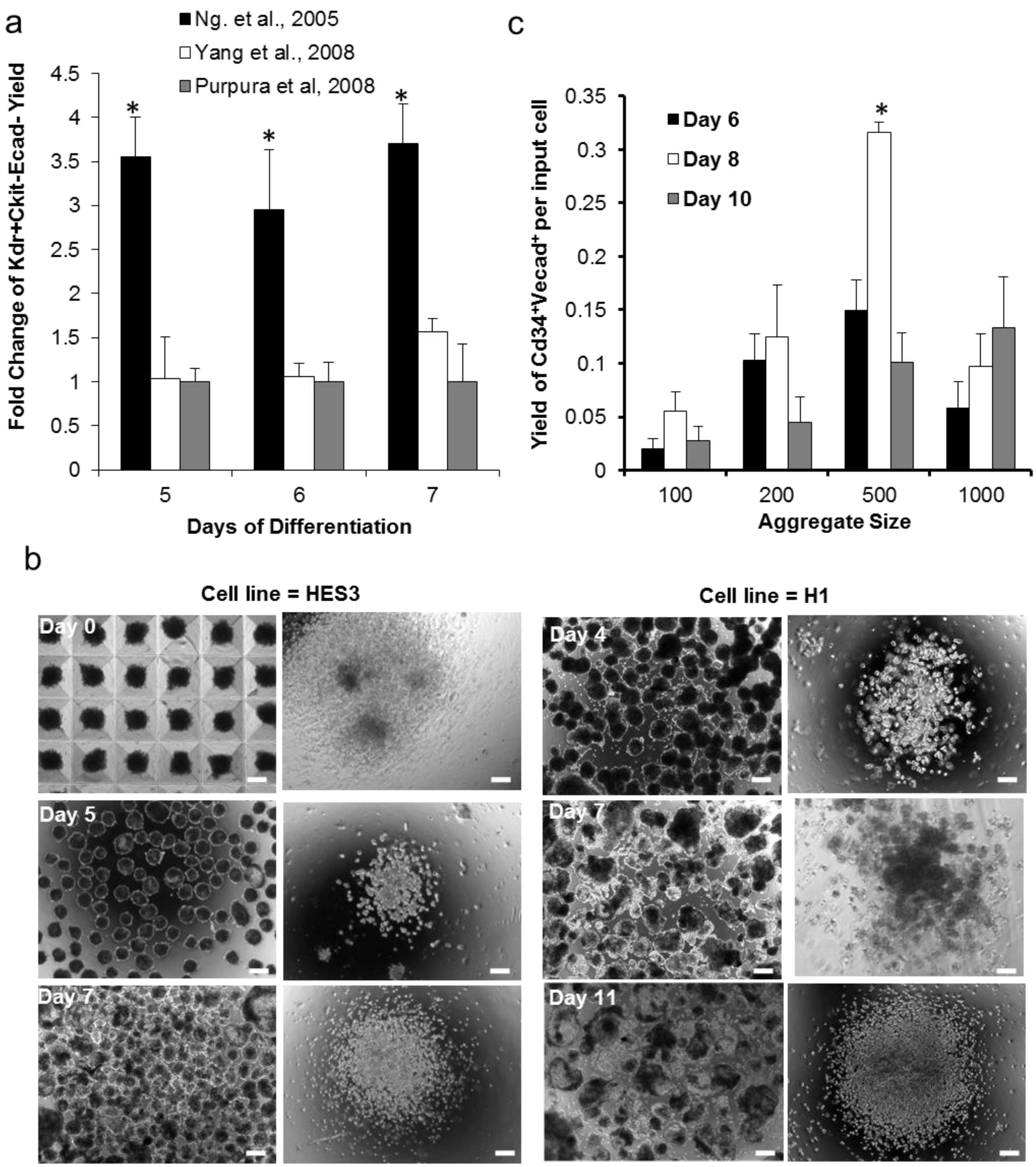

Supplementary Figure 1: Comparison of serum-free differentiation protocols. (a) KDR+ECAD-CKIT- expression of differentiating hPSCs among three differentiation protocols on day 5, 6 and 7, n = 3. Ng et al. uses BMP4, VEGF, bFGF & SCF [d0 –d7]; Yang et al. uses BMP4 [d0-d4], VEGF [d4-d7], bFGF [d4-d7], Activin A [d4-d7], Dickkopf [d4-d7]; Purpura et al. uses BMP4 [d0-d7], VEGF [d0-d5], Tpo [d0-d7] , n = 3 **(b)** Left column: **r**epresentative bright field images during differentiation of RUNX1-HES3 displaying uniform starting aggregate sizes (left column) and myeloid-lineage cells from hPSC-derived blood progenitors (CFU-GM [granulocyte-macrophage], CFU-M[macrophage], CFU-G [granulocyte]) (right column) **(b)** Right column: serum-free differentiation of H1 cells displaying bright field images of differentiating aggregates (left column) and **(**CFC generation (right column). **(c)** Yield of CD34+VECAD+ prospective HE cells per input hPSCs from aggregates initiated with 100, 200, 500, and 1000 cells , n = 3. Data are presented as mean ± SEM. Scale bar = 200 µm. Treatments that do not share asterisks are significantly different ($p \leq 0.05$, one-way ANOVA post hoc Tukey test). Related to **Figure 1.**

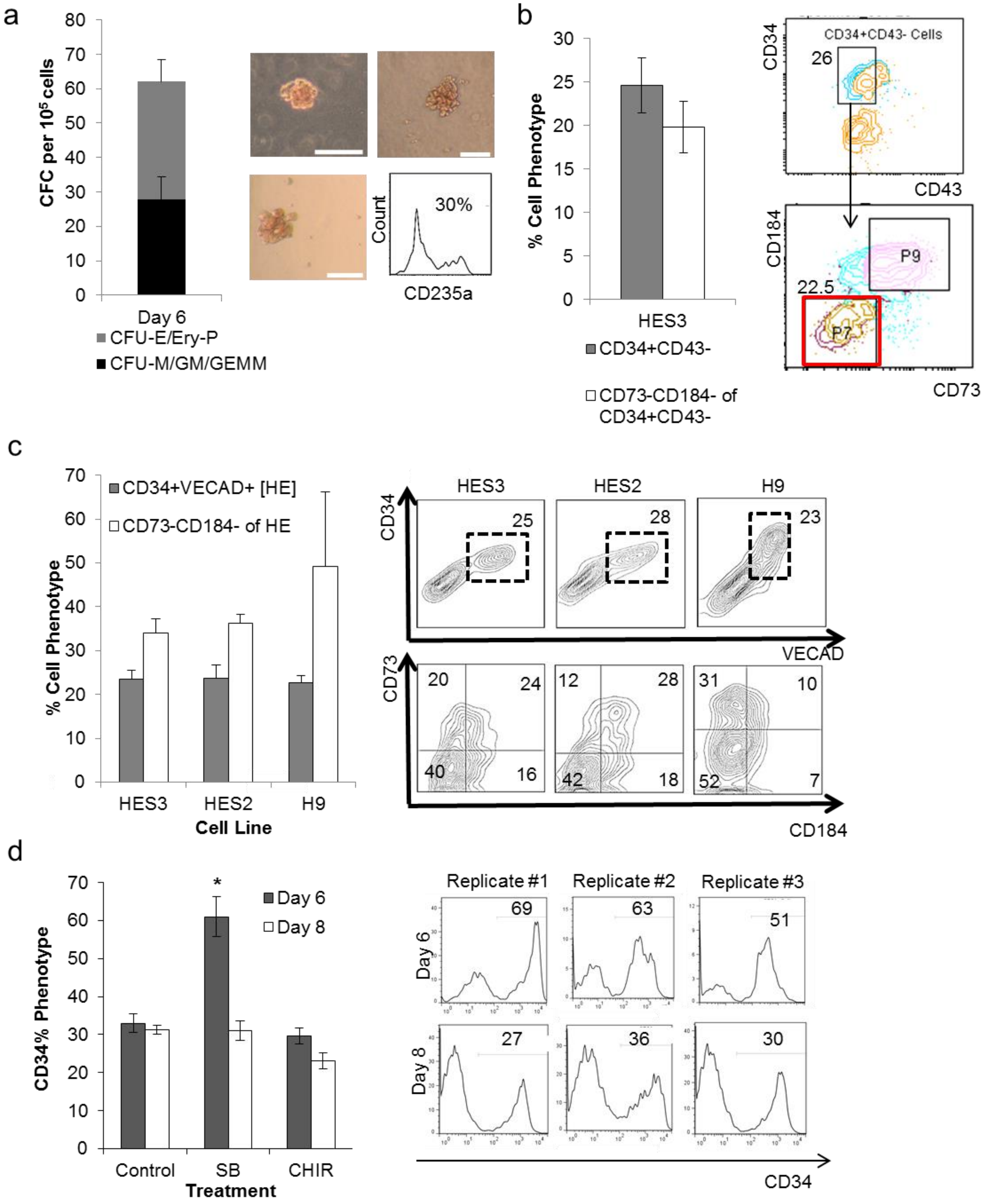
a
CFC per 10^5 cells
80
70
60
50
40
30
20
10
0
Day 6
CFU-E/Ery-P
CFU-M/GM/GEMM
Count
30%
CD235a
b
% Cell Phenotype
30
25
20
15
10
5
0
HES3
CD34+CD43-
CD73-CD184- of CD34+CD43-
CD34
CD34+CD43- Cells
26
CD43
CD184
P9
22.5
P7
CD73
c
70
60
50
40
30
20
10
0
CD34+VECAD+ [HE]
CD73-CD184- of HE
% Cell Phenotype
HES3
HES2
H9
Cell Line
CD34
25
28
23
VECAD
CD73
20
24
12
28
31
10
40
16
42
18
52
7
CD184
d
70
60
50
40
30
20
10
0
*
Day 6
Day 8
CD34% Phenotype
Control
SB
CHIR
Treatment
Replicate #1
Replicate #2
Replicate #3
Day 6
69
63
51
Day 8
27
36
30
CD34

Supplementary Figure 2: Characterization of hPSC-derived HE cells. **(a)** Enumeration of CFCs from day 6 hPSC-derived cell depict erythroid progenitors, n = 3 and representative bright field images of colonies displaying erythroid-lineage. Colonies isolated from methylcellulose cultures are positive for CD235a+ expression. **(b)** Phenotypic analysis for detection of CD34+CD43- and CD73-CD184- of CD34 +CD43- populations via flow cytometry, n = 3. **(c)** Phenotypic analysis for detection of CD34+VECAD+ (HE) expression and CD73-CD184- of CD34+VECAD+ expression across three parental hPSC lines – HES3, HES2 and H9 , n =3. Representative flow cytometry plots from day 8 hPSC-derived differentiating cells depicting CD34+VECAD+ induction (top) and CD73/CD184 induction (bottom) across three parental hPSC lines HES3, HES2 and H9. **(d)** CD34+ expression in control, SB431542 (SB)-treated, Chiron (CHIR)-treated hPSC-derived cells on day 6 and day 8, n =3 and flow cytometry plots displaying CD34+ expression across all replicates in SB-treated samples. Data are presented as mean ± SEM. Treatments that do not share asterisks are significantly different. Related to **Figure 2**.

a

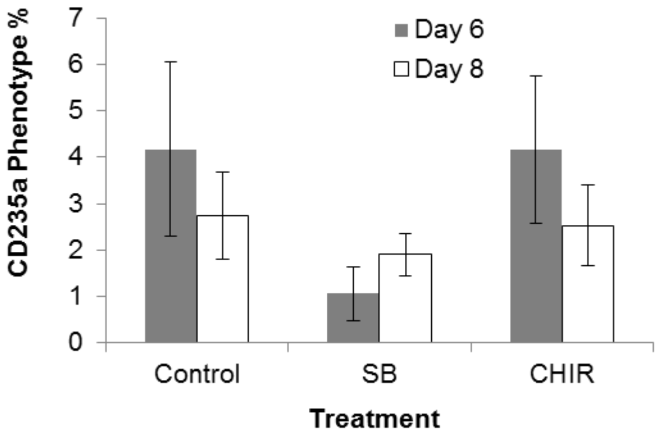

Supplementary Figure 3: CD235a expression in control, SB and CHIR-supplemented treatments. (a) CD235a expression; n= 3. Error bars represent standard error means. Related to **Figure 2**.

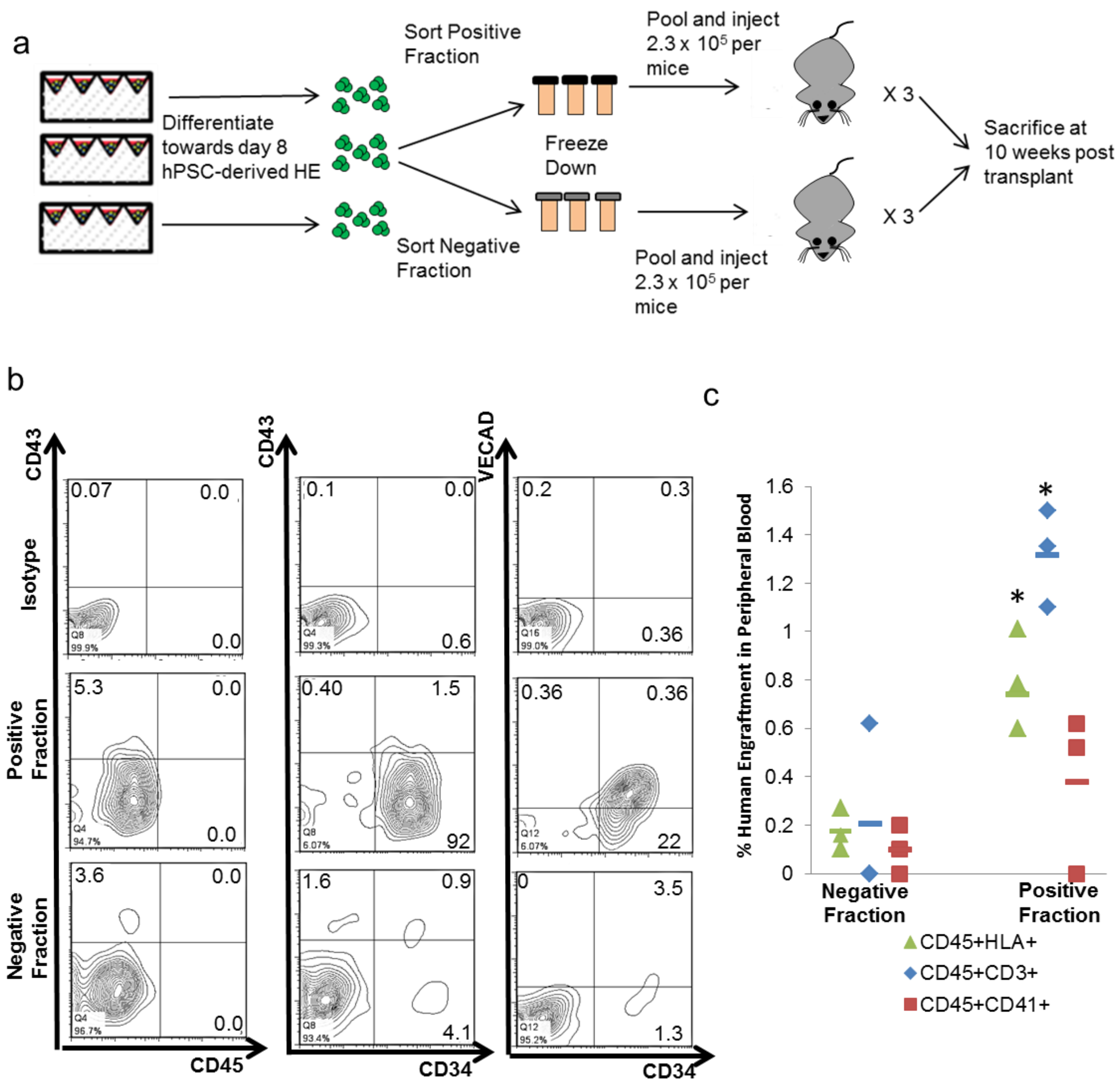

Supplementary Figure 4: Cellular phenotypes from in vivo studies. (a) Experimental layout for in vivo studies: hPSC-derived cells are differentiated towards HE cells and sorted into positive and negative fractions for injection into immunocompromised NSG mice. **(b)** Post-thaw analysis of isotype, positive and negative fraction injected into NSG mice. **(c)** Phenotypic analysis of human CD45+HLA+ (hematopoietic), CD45+CD3+ (T-cells) and CD45+CD41+ (megakaryocyte) expression in peripheral blood of NSG mice recipients 10 weeks post injection. Respective treatments that do not share asterisks are significantly different at $p \leq 0.05$ based on Students' T-test. Related to **Figure 2**.

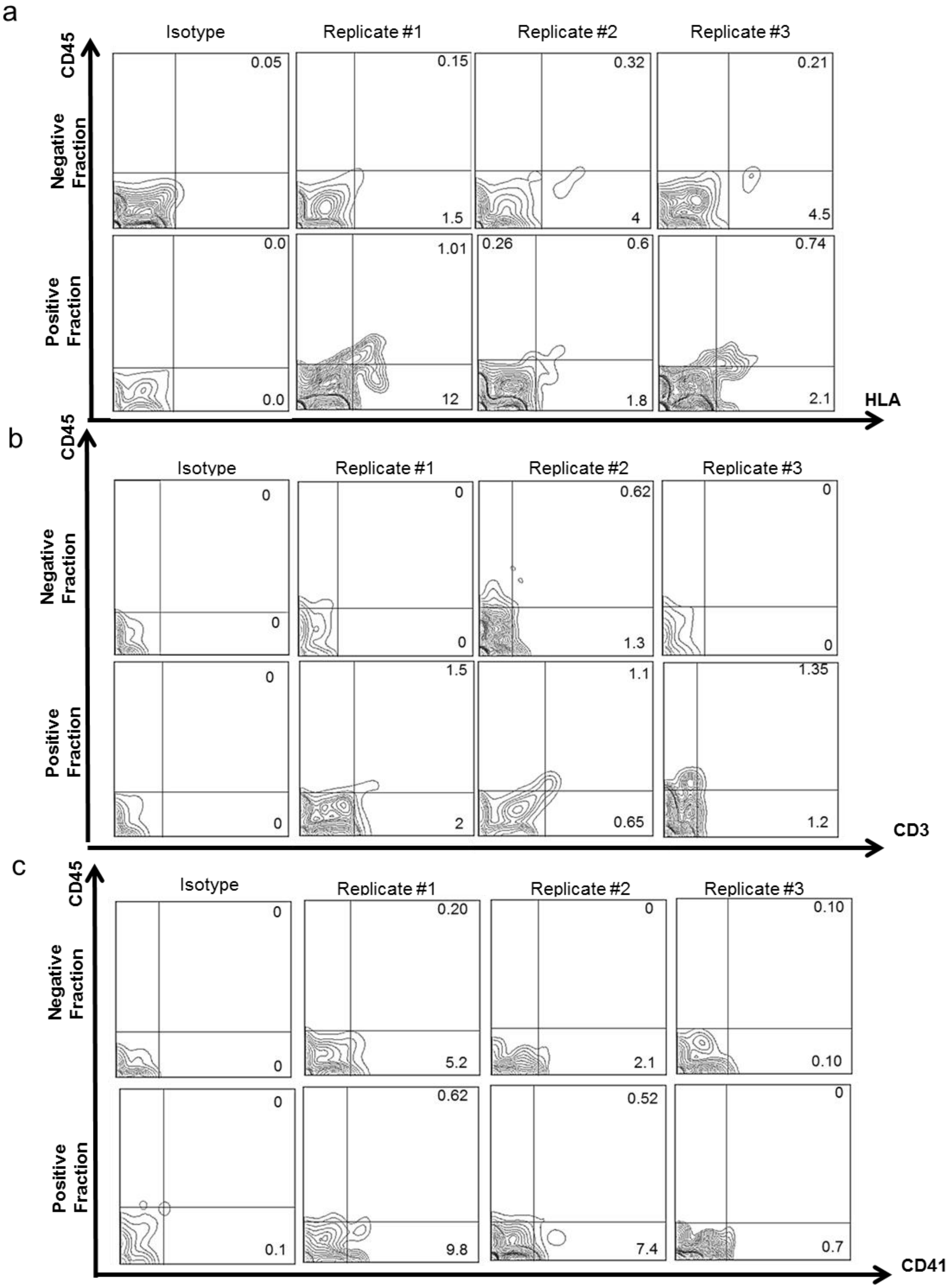

a
Isotype
Replicate #1
Replicate #2
Replicate #3
CD45
Negative Fraction
Positive Fraction
0.05
0.15
0.32
0.21
1.5
4
4.5
0.0
1.01
0.26
0.6
0.74
0.0
12
1.8
2.1
HLA
b
CD45
Isotype
Replicate #1
Replicate #2
Replicate #3
Negative Fraction
Positive Fraction
0
0
0.62
0
0
0
1.3
0
0
1.5
1.1
1.35
0
2
0.65
1.2
CD3
c
CD45
Isotype
Replicate #1
Replicate #2
Replicate #3
Negative Fraction
Positive Fraction
0
0.20
0
0.10
0
5.2
2.1
0.10
0
0.62
0.52
0
0.1
9.8
7.4
0.7
CD41

Supplementary Figure 5: **Flow cytometry plots for detecting human engrafted cells in peripheral blood of NSG mice recipients 10 weeks post injection. :** Flow cytometry plots for detecting human engrafted cells in peripheral blood of NSG mice recipients 10 weeks post injection. **(a)** CD45+HLA+ (Live cells analyzed: Isotype negative: 454, Replicate #1 negative: 193, Replicate #2 negative: 265, Replicate #3 negative: 155; Isotype positive: 216, Replicate #1: 170, Replicate #2: 217, Replicate #3: 105). **(b)** CD45+CD3+ (Live cells analyzed: Isotype negative: 472, Replicate #1 negative: 326, Replicate #2 negative: 93, Replicate #3 negative: 105; Isotype positive: 442, Replicate #1: 122, Replicate #2: 326, Replicate #3: 79) and (c) CD45+CD41+ (Live cells analyzed: Isotype negative: 210, Replicate #1 negative: 96, Replicate #2 negative: 105, Replicate #3 negative: 35; Isotype positive: 202, Replicate #1: 82, Replicate #2: 293, Replicate #3: 382). Data set is across all biological replicates. Related to **Figure 2.**

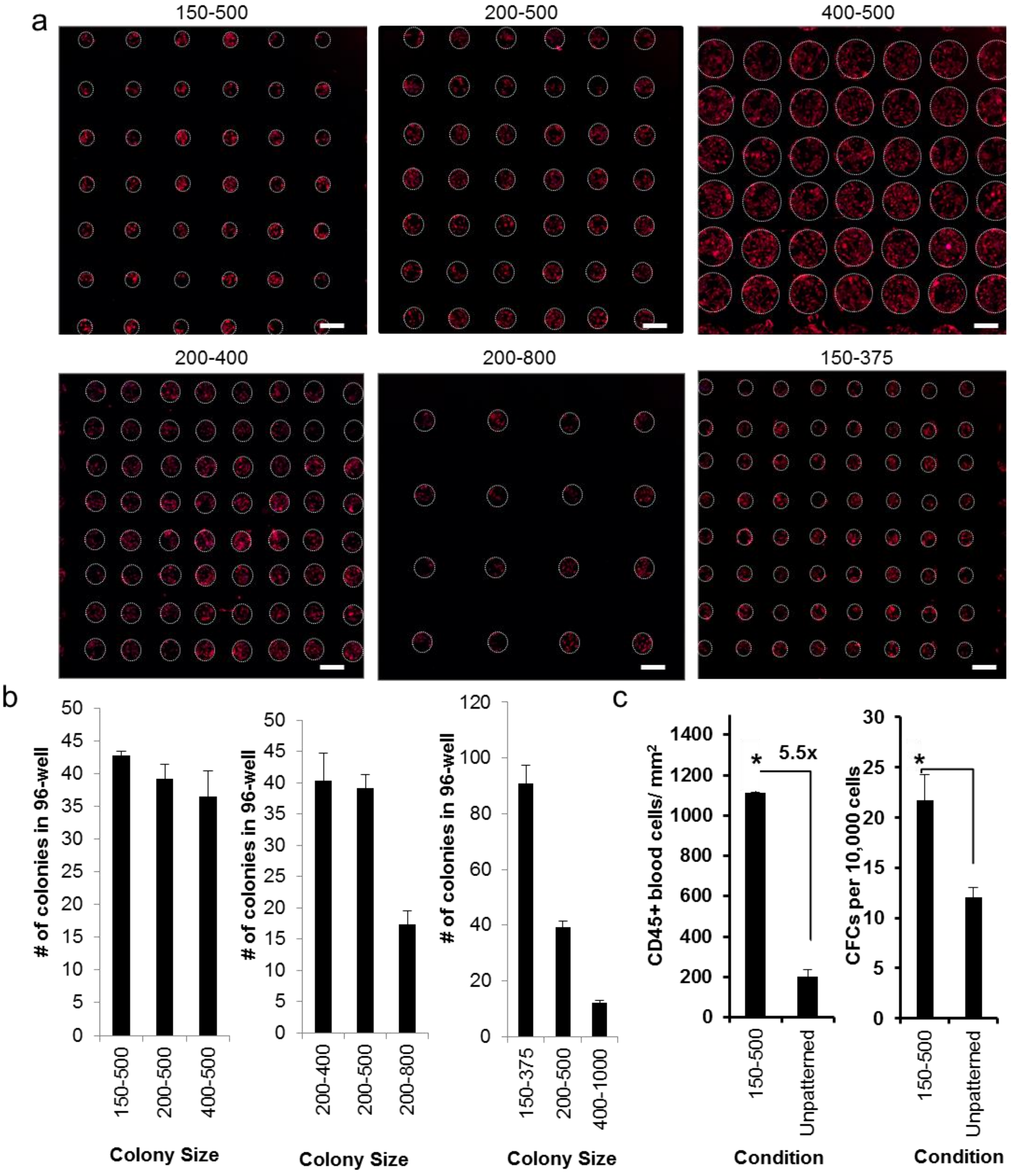
a
150-500
200-500
400-500
200-400
200-800
150-375
b
# of colonies in 96-well
Colony Size
150-500
200-500
400-500
200-400
200-800
150-375
400-1000
c
CD45+ blood cells/ mm²
5.5x
*
Unpatterned
Condition
CFCs per 10,000 cells

Supplementary Figure 6: Representation of day 8 seeded HE micropatterns. (a) Low magnification representative images of HE colonies (red- VE-Cadherin; blue – Hoechst). Colonies are outlined with white dotted lines. **(b)** Number of colonies present in one 96-well in increasing coverage, pitch and clustering treatments, n =3. Micropatterns are depicted as *diameter-pitch* (*diameter*: diameter of colony/ *pitch*: distance between colony centers; e.g.: 150-500). **(c)** CD45+ blood cell generation per surface area between 150-500 and non-micropatterned treatments, n = 3; and enumeration of CFCs from generated blood cells (in suspension) cells between 150-500 colonies and non-micropatterned conditions. Data are presented as mean ± SEM, n = 3. Related to **Figure 2**.

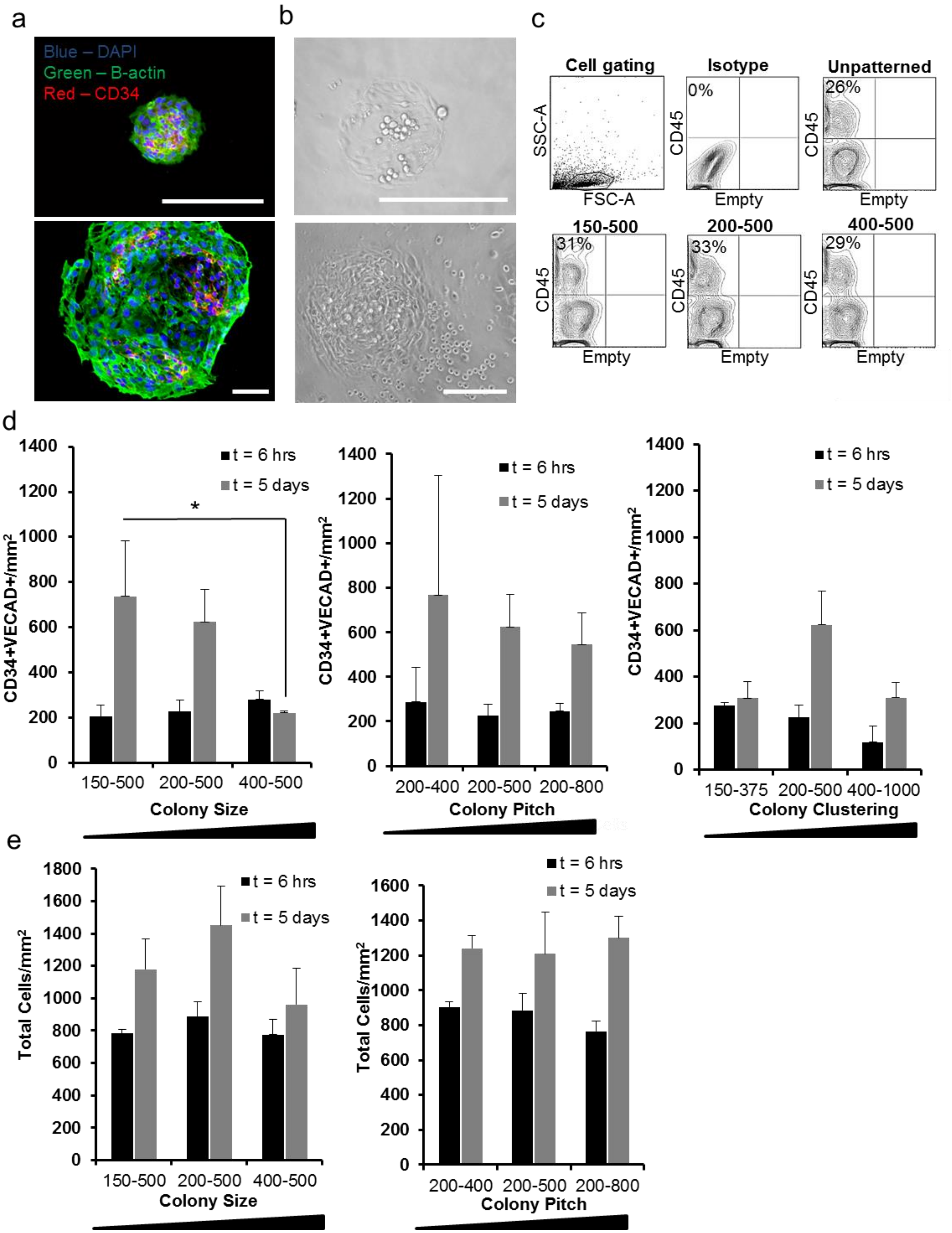
a
Blue – DAPI
Green – B-actin
Red – CD34
b
c
Cell gating
SSC-A
FSC-A
Isotype
0%
CD45
Empty
Unpatterned
26%
150-500
31%
200-500
33%
400-500
29%
d
t = 6 hrs
t = 5 days
CD34+VECAD+/mm²
*
150-500
200-500
400-500
Colony Size
200-400
200-800
Colony Pitch
150-375
400-1000
Colony Clustering
e
Total Cells/mm²

Supplementary Figure 7: Effect of micropatterned HE colonies on blood induction. (a) Representative fluorescent image of 150 µm and 400 µm diameter patterned hPSC-derived colonies after 5 days of hematopoietic induction (green – B-actin; red – CD34, blue - DAPI). **(b)** Representative brightfield images of day 8 150 µm and 400 µm patterned colonies giving rise to CD45+ 'budding' blood cells after five days of hematopoietic induction. **(c)** Representative flow cytometry plots of CD45+ expression in differentiated blood cells at t = 5 days, n = 3 **(d)** CD34+VECAD+ induction per cultured area among micropatterns at t = 6 hours and t = 5 days in increasing colony size, pitch, and clustering., n = 3. **(e)** Total cells patterned per cultured area in increasing colony size and pitch, n =3. Data are presented as mean ± SEM. Treatments that are joined by asterisks are significantly different at $p \leq 0.05$ using Student's t-test. Scale bar = 200 µm. Related to **Figure 4**.

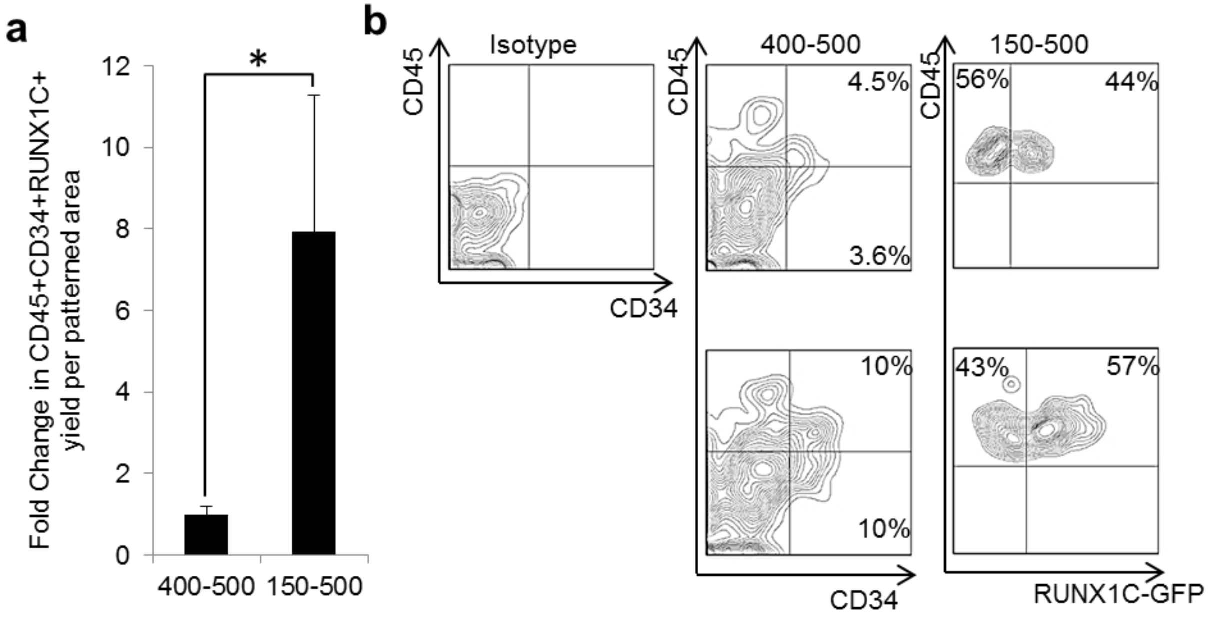

Supplementary Figure 8: Micropatterned colony size and density influence hematopoietic induction in differentiating PSCs. (a) Fold change expression of CD45+CD34+RUNX1C+ of blood cells from 150-500 micropatterned colonies compared to 400-500 micropatterned colonies, n =3. Treatments that share asterisks are significantly different ($p \leq 0.05$, Student's t-test). **(b)** Representative flow cytometry plots. Statistical significance was computed via Student's t-test. All error bars represent standard deviation. n = 3. Related to **Figure 3.**

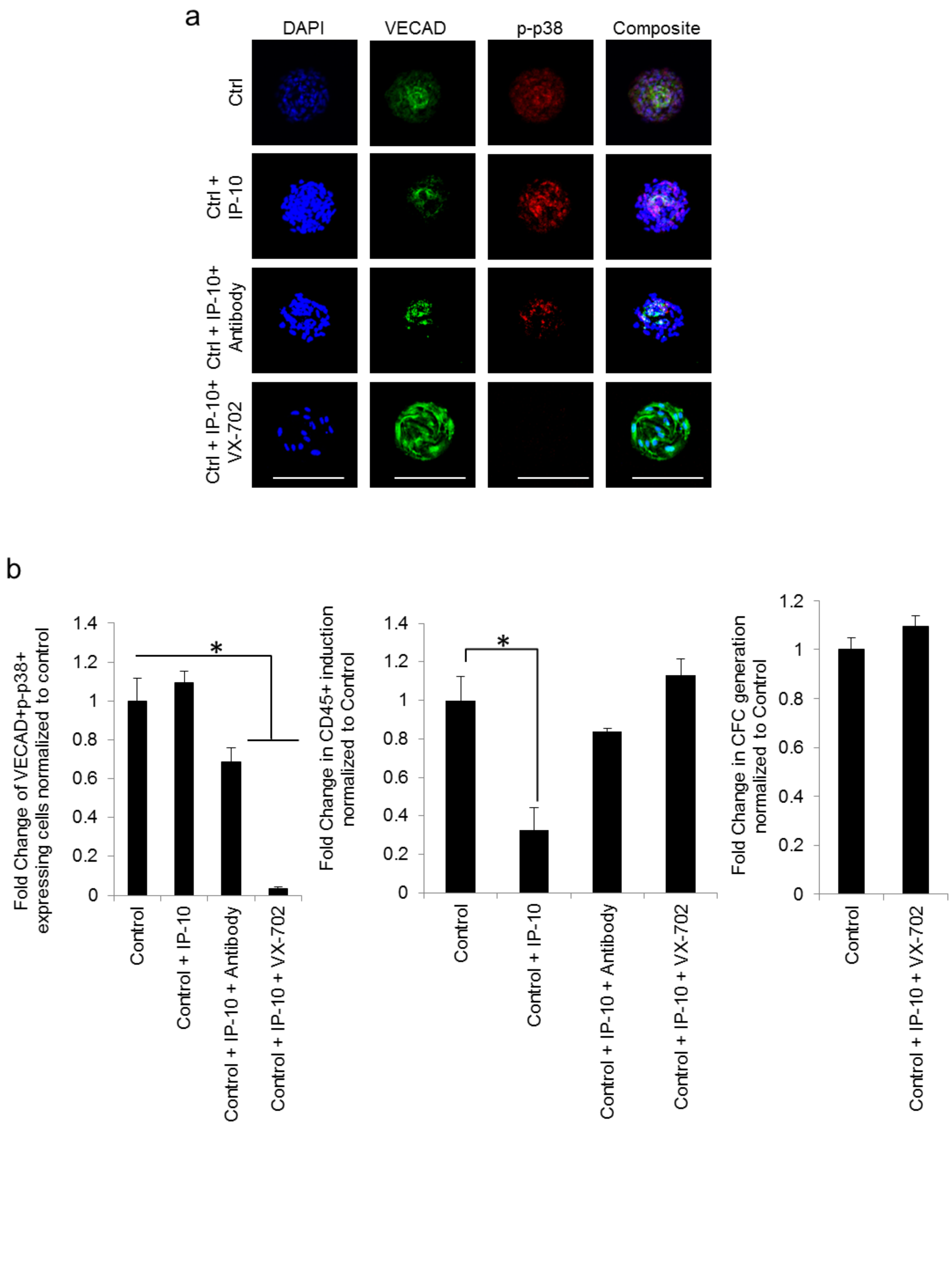

a
DAPI
VECAD
p-p38
Composite
Ctrl
Ctrl + IP-10
Ctrl + IP-10+ Antibody
Ctrl + IP-10+ VX-702
b
Fold Change of VECAD+p-p38+ expressing cells normalized to control
1.4
1.2
1
0.8
0.6
0.4
0.2
0
*
Control
Control + IP-10
Control + IP-10 + Antibody
Control + IP-10 + VX-702
Fold Change in CD45+ induction normalized to Control
1.4
1.2
1
0.8
0.6
0.4
0.2
0
*
Control
Control + IP-10
Control + IP-10 + Antibody
Control + IP-10 + VX-702
Fold Change in CFC generation normalized to Control
1.2
1
0.8
0.6
0.4
0.2
0
Control
Control + IP-10 + VX-702

Supplementary Figure 9: Interferon gamma induced protein 10 (IP-10) reductions of CD45+ hPSC-derived blood progenitor cells can be rescued by inhibiting the p38 MAPK pathway. (a) Representative fluorescent image of 150 µm diameter patterned hPSC-derived colonies after 5 days of hematopoietic induction among control (Ctrl),Ctrl + IP-10, Ctrl + IP-10 + anti-CXCR3 antibody, Ctrl + IP-10 + VX-702 (p38 MAPK inhibitor) treatments (green – VECAD; red – p-p38; blue – DAPI). Scale bar = 100 µm. **(b)** Fold change in induction normalized to control of VECAD+p-p38+ expressing cells, n = 2 (n ≥ 300 micropatterned colonies), CD45+ expressing cells, n ≥ 3 and CFC generation, n ≥ 3. Treatments that share asterisks are significantly different (p≤ 0.05, one-way ANOVA with post-hoc Tukey test). All error bars represent standard error mean. Scale bar = 150 µm. Related to **Figure 3.**

a

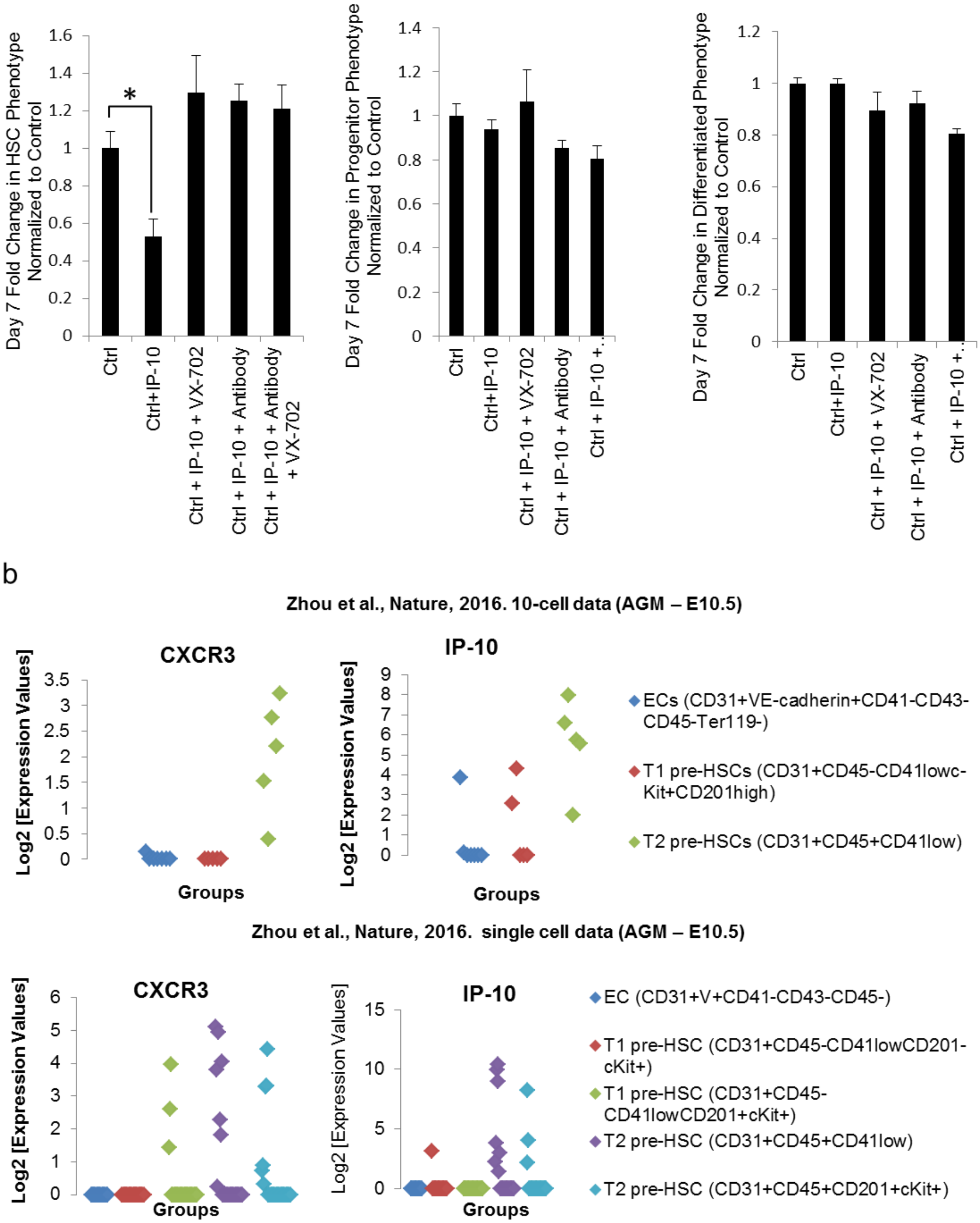
Day 7 Fold Change in HSC Phenotype Normalized to Control
Day 7 Fold Change in Progenitor Phenotype Normalized to Control
Day 7 Fold Change in Differentiated Phenotype Normalized to Control
Ctrl
Ctrl+IP-10
Ctrl + IP-10 + VX-702
Ctrl + IP-10 + Antibody
Ctrl + IP-10 + Antibody + VX-702
Ctrl + IP-10 +...
*
b
Zhou et al., Nature, 2016. 10-cell data (AGM – E10.5)
CXCR3
IP-10
Log2 [Expression Values]
Groups
ECs (CD31+VE-cadherin+CD41-CD43-CD45-Ter119-)
T1 pre-HSCs (CD31+CD45-CD41lowc-Kit+CD201high)
T2 pre-HSCs (CD31+CD45+CD41low)
Zhou et al., Nature, 2016. single cell data (AGM – E10.5)
EC (CD31+V+CD41-CD43-CD45-)
T1 pre-HSC (CD31+CD45-CD41lowCD201-cKit+)
T1 pre-HSC (CD31+CD45-CD41lowCD201+cKit+)
T2 pre-HSC (CD31+CD45+CD41low)
T2 pre-HSC (CD31+CD45+CD201+cKit+)

Supplementary Figure 10: Interferon gamma induced protein 10 (IP-10) treatment of CD34+ sorted cord blood cells decreases hematopoietic stem cell (HSC) phenotype. (a) Day 7 fold change in induction normalized to control of HSC phenotype [CD34+CD90+CD45RA-CD38/CD34+CD90+CD49f +CD45RA-CD38-], progenitor phenotype [CD34+CD90+CD45RA+/CD34+CD45RA+CD90-/CD34-CD45RA-CD90-] and differentiated phenotype [CD34-], n = 4. **(b)** Meta-analysis of RNA-seq data displaying CXCR3 and CXCL10 (IP-10) expression from hematopoietic fractions in embryonic day 10.5 AGM tissues containing 10-cells or single-cell analysis. Treatments that share asterisks are significantly different ($p \leq 0.05$, one-way ANOVA using post-hoc Tukey test). All error bars represent standard error mean. Related to **Figure 3.**

Supplementary Tables

Gene	Sequence (5' to 3')		Accession #	Product size (bp)
NOTCH1	Fwd	CAACGCCTACCTCTGCTTCT	NM_017617.4	158
	Reverse	ACATGCTCCCTGTGTAGCC		
EPHRINB2	Fwd	ACG ACA CTT TGG TTT ATG CGG TGC	NM_004093.3	116
	Reverse	AAG AGG TCT GCC GTA TGT GCT TCA		
KDR	Fwd	GCATGGAAGAGGATTCTGGA	NM_002253.2	109
	Reverse	CTGATTCCTGCTGTGTTGTCA		
DLL4	Fwd	CCT GCA TTG TGA ACA CAG CAC CTT	NM_019074.3	149
	Reverse	ACC TGT CCA CTT TCT TCT CGC AGT		
JAG1	Fwd	CAACCGTGCCAGTGACTATTTCTGC	NM_000214.2	250
	Reverse	TGTTCCCGTGAAGCCTTTGTTACAG		
NR2F2	Fwd	GCCATAGTCCTGTTCACCT	NM_001145157.1	83
	Reverse	GCACACTGAGACTTTTCCTG		
MYB	Fwd	CATTTGATCCGCATCCCCTG	NM_005375.3	154
	Reverse	TCAAAAGTTCAGTGCTGGCC		
TAL1	Fwd	ACTTGCCTTCCTAAGCCTGT	XM_017002193.1	179
	Reverse	CATTCACTCGCCAGCATGAA		
GAPDH	Fwd	GTTTACATGTTCCAATATGATTCCAC	NM_001289746.1	104
	Reverse	TGGAAGATGGTGATGGGATT		

Supplementary Table 1. Primers and corresponding accession numbers used for quantitative RT-PCR
